# Operational demands on pre-hospital emergency care for burn injuries in a middle-income setting: a study in the Western Cape, South Africa

**DOI:** 10.1186/s12245-017-0128-9

**Published:** 2017-01-25

**Authors:** Rachel L. Allgaier, Lucie Laflamme, Lee A. Wallis

**Affiliations:** 1 Department of Public Health Sciences, Karolinska Institutet, Stockholm, Sweden; 2 Division of Emergency Medicine, Department of Interdisciplinary Health Sciences, Stellenbosch University, Tygerberg, South Africa; 3 University of South Africa, Pretoria, South Africa

**Keywords:** Pre-hospital emergency care, Healthcare systems, Burns

## Abstract

**Background:**

Burns occur disproportionately within low-socioeconomic populations. The Western Cape Province of South Africa represents a middle-income setting with a high rate of burns, few specialists and few burn centres, yet a well-developed pre-hospital system. This paper describes the burn cases from a viewpoint of operational factors important to pre-hospital emergency medical services.

**Methods:**

A retrospective, cross-sectional study of administrative and patient records was conducted. Data were captured for all pre-hospital burn patients treated by public Emergency Medical Services over a continuous 12-month period. Data were captured separately at each site using a standardised data collection tool. Described categories included location (rural or urban), transport decision (transported or remained on scene), age (child or adult) and urgency (triage colour).

**Results:**

EMS treated 1198 patients with confirmed burns representing 0.6% of the total EMS caseload; an additional 819 potential burn cases could not be confirmed. Of the confirmed cases, 625 (52.2%) were located outside the City of Cape Town and 1058 (88.3%) were transported to a medical facility. Patients from urban areas had longer mission times. Children accounted for 37.5% (*n* = 449) of all burns. The majority of transported patients that were triaged were yellow (*n* = 238, 41.6% rural and *n* = 182, 37.4% urban).

**Conclusions:**

Burns make up a small portion of the EMS caseload. More burns occurred in areas far from urban hospitals and burn centres. The majority of burn cases met the burn centre referral criteria.

## Background

In high-income countries, pre-hospital emergency medical services (EMS) aim to respond to the ill and injured urgently and, when warranted, transport patients to an appropriate centre for definitive care. However, for most low- or middle-income countries (LMICs), where more than 90% of all deaths due to injury occur, formal pre-hospital systems do not exist [1–3]. The Disease Control Priorities in Developing Countries project suggests that in LMICs, as much as 10% of all injury deaths and 11% of disabilities from injuries could be addressed by strengthening the development of emergency care systems [4]. This was further advocated in the May 2007 World Health Assembly Resolution 60.22 [2].

Throughout South Africa, a middle-income country, the pre-hospital EMS system is varied: services in some parts of the country are well developed and employ many highly trained and experienced practitioners while services elsewhere are more rudimentary and utilise mostly basic providers. Hospital services in the country, like those in many LMICs, are unequally distributed not only geographically but also socioeconomically [1, 5]. Because of this, the pre-hospital system struggles to reach patients, both in rural areas due to the vast topography, as well as in urban areas that are interspersed with poorly demarcated roads and houses located within dilapidated informal settlements [1]. Though this inequity is being addressed [6], the problem is further compounded by a lack of specialised hospital services, which when combined with misdistribution of hospitals, inhibits good patient outcomes [7]. With this variability present in hospital services, the role of pre-hospital EMS in the wider healthcare system becomes essential toward ensuring favourable patient outcomes.

Understanding the impact that specific clinical conditions have on EMS may help improve planning and service delivery [8] and may inform the planning of emerging pre-hospital healthcare services throughout South Africa and in other LMIC settings [4]. In this paper, we focus on burn injuries, a general problem in LMICs [9] and in South Africa in particular [10]. There are few burn specialists in the country and only a small number of burn centres located in big cities. Most reports about burns in LMICs, including those from South Africa, emanate from hospital data within urban centres [11]. While valuable, this information only presents part of the scope of burns; [12] the burden of burns on pre-hospital EMS—and the service delivery afforded to them—is unknown. This paper aims to describe the operational services provided within the public, pre-hospital healthcare service as a result of burn injury calls. To offer caseload and an operational perspective, burn services are compared to other types of acute pre-hospital traumas (e.g. bites and stings, gunshot wounds and motor vehicle incidents).

## Methods

This was a retrospective, cross-sectional study based on administrative and clinical records from the 2011 calendar year.

### Setting and population

The Western Cape, one of South Africa’s nine provinces, runs a government-led EMS (WCG EMS). This service mostly caters to the uninsured 80% of the population, of which approximately 10% utilise the service annually [6]. WCG EMS transports patients to 34 district, four regional and three central public hospitals. One adult and one paediatric burn unit are located within the City of Cape Town. In 2011, WCG EMS had a total of 42 ambulance bases (38 rural), 145 operational ambulances and an operational workforce of 1373. Of the personnel, 52% were basic life support (BLS), 39% intermediate life support (ILS), and 9% advanced life support (ALS). All personnel operated under a protocol-driven system, dependent upon their scope of training, with no on-scene physician oversight.

The WC’s population was 5.8 million [13] in 2011 with 25.1% children <15 years [14]. Approximately 36% of the population (2.1 million) were living outside the urban City of Cape Town [15] in five rural municipalities covering 98% of the province (average 16 people/km^2^).

### Data sources and classification

Burn cases were identified as follows: operational information about the location, type and timing of each emergency call (hereinafter the ‘call’ was scrutinised from routinely captured electronic spreadsheets. Calls were then electronically filtered for ‘trauma—burns and corrosives’ or ‘paediatric—burns and corrosives’ as categorised by WCG EMS personnel and were classified as ‘reported burns’ for the purposes of this study. Patient report forms containing demographic and clinical information about the patient as documented on paper by EMS at the scene of the emergency were inspected. WCG EMS triage patients using the South African Triage Scale (SATS), a four-level system where green represents the least and red the most urgent cases; [16] SATS has at its core, the Triage Early Warning Score (TEWS), a physiologic scoring component from zero to 17 which is also recorded by EMS [17, 18]. Call centre statistics were used to provide an overview of the total EMS caseload since some operational spreadsheets could not be obtained. This data source also provided information about additional traumas for comparisons about caseload and transport times, etc. Missing data were reported as missing.

Cases were categorised as urban if the call was dispatched within the City of Cape Town. All other calls were considered rural for this study. Operational timestamps were calculated as follows and measured against WCG EMS targets:

$$ \begin{array}{l}\mathrm{mission}\kern0.3em \mathrm{time}=\mathrm{free}\kern0.3em \mathrm{time}\kern0.1em {\textstyle \hbox{-}}\kern0.1em \mathrm{incident}\kern0.3em \mathrm{time},\kern0.5em \\ {}\mathrm{scene}\kern0.3em \mathrm{time}=\mathrm{depart}\kern0.3em \mathrm{scene}\kern0.3em \mathrm{time}\kern0.1em {\textstyle \hbox{-}}\kern0.1em \mathrm{arrive}\kern0.3em \mathrm{on}\kern0.3em \mathrm{scene}\kern0.3em \mathrm{time},\mathrm{and}\kern0.5em \\ {}\mathrm{response}\kern0.3em \mathrm{time}=\mathrm{scene}\kern0.3em \mathrm{time}\kern0.1em {\textstyle \hbox{-}}\kern0.1em \mathrm{incident}\kern0.3em \mathrm{time}.\end{array} $$

A priority level for each call had been determined at the call centre by the call taker using a standardised Priority Based Dispatch tool. Priority 1 response time targets were <15 and <40 min for calls in urban and rural areas, respectively, while priority 2 response time targets remain <30 and <60 min for calls in urban and rural areas, respectively. WCG EMS aims to achieve these targets for 90% of all calls. Disposition was determined using the South African Burn Society’s criteria for referral to a burn centre (Table 1) [19].

**Table 1:** Minimum criteria for transfer to a South African burn centre

Burn injury patients meeting any of the following criteria should be transferred to a burn centre	
• <1 year of age • 1–2 years old with burns >5% TBSA • All third degree burns • >2 years of age with partial-thickness burns >10% TBSA • Special areas (face, hands, feet, genitalia, perineum or major joints) • Electrical burns, including lightning burns • Chemical burns • Inhalation injury resulting from fire or scald burns • Circumferential burns of the limbs or chest • Pre-existing medical disorders that could complicate management, prolong recovery or affect mortality • Concomitant trauma • Paediatric cases where child abuse is suspected • Treatment requirements exceeding the capabilities of the referring centre • Septic burn wounds	

This table has been adapted with permission after Karpelowsky et al. modified the Australian and New Zealand Burn Association protocol [19].

*TBSA* total body surface area

### Data collection

**Fig. 1:**
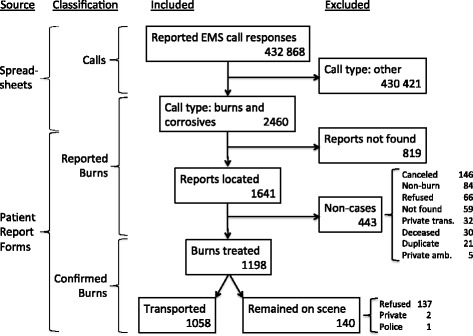
2011 burn data collected from Emergency Medical Services in the Western Cape

Data were retrospectively collected at EMS bases by the first author and trained medical students. All burns reported to EMS between 1 January and 31 December 2011 were included. Data collected from operational spreadsheets included all timestamps from call receipt to call completion. Found patient report forms were reviewed to confirm the presence of a burn injury. Patient report form data were captured and transcribed as documented to the operational spreadsheets to supplement each call’s data. Cases were excluded if unconfirmed or when WCG EMS was not involved, etc. (Fig. 1). Confirmed cases where patients refused transport or were transported by police or private ambulance after receiving treatment were not considered ‘transported cases’.

### Data treatment and analysis

Data were stored, collated and analysed using Microsoft Excel (Redmond, WA, USA). Demographic, injury and clinical data were reported using means or medians with inter-quartile ranges (IQR) and included caseload, time, urgency, referral and resources. Data within categories (e.g. location; urban vs rural, age: child vs adult) were described in relation to EMS’s involvement (e.g., time, resources).

## Results

### Caseload

A total of 432,868 emergency calls were captured in 369 (73.2%) spreadsheets, with approximately 0.6% (*n* = 2460) of calls reported as burns. Regarding patient report forms, 1641 were obtained; the remaining were not found (*n* = 599) or not searched for (*n* = 220) due to EMS base impediments (Fig. 1). A total of 1198 burns could be confirmed by patient report form, 37.5% (*n* = 449) of which were children (Table 2). Just over half (52.2%) of all cases were from rural areas. Of all patients, 88.3% received transport to a healthcare facility. The main reasons for unconfirmed burns (*n* = 443) and transported case exclusion (*n* = 140) are outlined in Fig. 1.

**Table 2:** Confirmed burn transport decisions by location and age (Western Cape Province, 2011)

		Transported		Remained on scene	
Age group	Number	Rural	Urban	Rural	Urban
Children^a^	449	221	194	11	23
Adults	749	351	292	42	64
Total	1198	572	486	53	87

^a^<13 years

From the WC call centre statistics for the study period, 26.9% of calls were for trauma and of these, 2.9% were burns. To compare, gunshot wounds, and bites and stings accounted for 1.2 and 2.1% of traumas, while transport-related injuries (e.g. bus/taxi, cyclist, motorcyclist, pedestrian, transport other, truck/heavy vehicle), light motor vehicle incidents and other weapon-related injuries were responsible for 8.7, 8.9 and 41.7% of trauma calls, respectively.

### Time

The median total mission time for transported burn calls was 69.0 min, with 50% of transported calls taking between 50.0 and 96.4 min. This was similar to other trauma calls with the shortest median time being 66.8 (IQR 45.7–94.5) min for bites and stings, and the longest being gunshot wounds (median 79.2, IQR 59.6–100.9 min). For transported, urban burn cases, an average of 15.8 more minutes was required than for transported, rural cases. In fact, this was true for all trauma types with an average of 17.1 more minutes required in urban areas. Median scene time accounted for 13 min in urban versus 11 min in rural areas, and 12.5 min for P1 and 11.5 min for P2 calls. Furthermore, in urban areas, 28.9 and 45.5% of cases met the priority 1 (<15 min) and priority 2 (<30 min) response time targets while in rural areas, 92.1 and 92.7% met the priority 1 (<40 min) and priority 2 (<60 min) targets.

### Urgency

Burn size and depth were to be used to calculate injury severity; however, these data were missing from 838 (70.0% size) and 539 (45.0% depth) patient report forms. As a proxy, triage (i.e. SATS) and TEWS values were used to identify the urgency of cases. Overall, 68.3% (*n* = 818) of patients were triaged green or yellow denoting low urgency, while 14.8% were triaged orange or red (Table 3). The remaining patients’ triage colours were not specified and accounted for the majority of patients that remained on scene (54.7% rural and 66.7% urban). No patients that remained on scene were triaged orange or red. The transported patients had variable TEWS values; however, all patients that remained on scene had TEWS values less than four, indicating low clinical urgency.

**Table 3:** Confirmed burn transport decisions by location, stratified by urgency and criteria for burn centre referral (Western Cape Province, 2011) [19]

Urgency/referral		Total	Transported		Remained on scene	
	Category	1198	Rural 572	Urban 486	Rural 53	Urban 87
		*n*	%	%	%	%
Triage	Green	386	35.5	29.2	37.7	24.1
	Yellow	432	41.6	37.4	7.5	9.2
	Orange	126	8.6	15.8	0	0
	Red	51	4.7	4.9	0	0
	Unspecified	203	9.6	12.6	54.7	66.7
Triage Early Warning Score	0	63	6.1	4.5	7.5	2.3
	1	187	15.9	16.7	11.3	10.3
	2	285	25.5	23.0	24.5	16.1
	3	217	21.5	18.5	0	4.6
	4+	254	22.7	25.5	0	0
	Unspecified	192	8.2	11.7	56.6	66.7
Burn centre referral criteria met	Yes, by body part	379	31.3	34.4	22.6	24.1
	Yes, by other criteria	273	19.8	25.9	17.0	28.7
	No	490	45.3	34.8	52.8	39.1
	Undetermined	56	3.7	4.9	7.5	8.0
			100	100	100	100

### Referral

The South African Burn Society’s referral criteria (Table 1) were used to determine *a* posteriori, which burns required referral to a dedicated burn centre (Table 3). For transported patients, 51.1% (*n* = 292) rural and 60.3% (*n* = 293) urban had burn injuries that met the burn centre referral criteria, compared to 39.6% (*n* = 21) rural and 52.8% (*n* = 46) urban with injuries that met the referral criteria, yet remained on scene. In total, 4.7% (*n* = 56) of cases did not provide enough information to determine whether referral criteria were met.

### Resources

Regarding human resources, the majority (62.5%) of ambulances treating and/or transporting burn patients had at least one ILS or ALS provider on-board; BLS-only crews made up 12.1% and the rest were undocumented. For burns meeting the burn centre referral criteria, 65.5% of patients were treated by either an ILS or ALS provider.

## Discussion

This study shows that in the Western Cape, EMS received a relatively small number of calls for burn injuries over the study year. About one in two burns occurred outside the City of Cape Town and had shorter mission times than urban cases. Just over half of all burns met the burn centre referral criteria.

In total, EMS attended approximately 3.2 burn cases per day in the WC. This is an underestimate of the burden when missing reports are considered. The vast majority of burns in this study received transport to a medical facility. No previous literature specific to burn patients’ arrival at hospital or transfer to burn units is available, nor is pre-hospital data about other trauma types available for comparison. Western Cape, hospital-based studies report between 280–360 adults [20] and approximately 1200 paediatric burn patients admitted to the two dedicated burn units, annually [21].

Priority levels assigned to calls by EMS call takers are a measure of perceived urgency and are used to monitor and evaluate response times [22]. While the utility of these measurements are debatable, they offer insight into service provision and allow for comparisons across trauma types within the system. Urban burn cases did not meet either targets, but rural burn cases met both priority 1 and 2 targets. Rural patients may have been transported to lower-level clinics or hospitals closer to where the incidents occurred, thereby reducing response times. It is impossible to determine from this study whether burn injuries as a trauma group do not fit well with the response time targets, or whether the response time targets are too ambitious in urban areas, for example. However, since the median total mission times were similar for other trauma types in both urban and rural locations, the data suggest ambulance crews are overall, taking comparable amounts of time for burns as other trauma cases. Still, an improvement in urban response time performance is required [23]. The times reported in this study take into consideration only the amount of time EMS is involved. Unfortunately, additional delays in presentation exist and have been shown to increase the length of stay in hospital [24] and negatively impact on survival [25]. These are usually due to lack of access and delays due to self-treatment, consultation with traditional healers and transport costs [26]. The focus of EMS is always to react with urgency once a call has been placed requesting emergency services; narrowing the time between the incident and the call will take significant improvements in public health awareness.

Approximately one in three patients were triaged green; however, according to SATS, all burns should at least be triaged yellow, by discriminator [16, 27]. This likely reflects a lack of understanding about triaging or assessing burns. All patients triaged orange or red or with a TEWS value >3 were transported which suggests that more severe burns, as determined by EMS personnel, were rightfully transported to receive emergency care, as intended by SATS protocols. Furthermore, there is a direct relationship between dispatched priority and SATS colour (i.e. measured urgency), as expected.

Even with a large amount of missing injury data, just over half of all burn injuries met the burn centre referral criteria. Interestingly, burns were not perceived to be urgent by EMS personnel according to the assigned triage category, yet they were severe enough to warrant referral to a burn centre. Some non-urgent (i.e. non-time sensitive) burns may be complicated due to burn site (e.g. hand, face) and therefore require expert care in a burn unit. Alternatively, burn centre referral criteria may not be a good indicator of burn urgency or severity in pre-hospital care. The nuances of these interpretations create complications for pre-hospital healthcare treatment and transport decisions; *when* to transport a patient to a burn centre is not indicated in the referral criteria, for pre-hospital or in-hospital patients. Furthermore, whether transport to a burn centre was attempted for these cases is not known as EMS first transport patients to the closest, most appropriate healthcare facility. This referral pathway is common in many countries and has not been shown to negatively impact patient outcomes [28].

The pre-hospital providers’ level of training determines the scope of care they may provide according to the severity of the injury. EMS aims to provide a minimum of ILS care for all calls requiring such services. In this study, 48% of EMS personnel were ideally trained to respond to burns (i.e. ILS and above) and responded to at least 62.5% of the calls while 25.4% were unknown. It is difficult to determine whether ‘adequate’ care was provided due to the lack in documentation about both the provider as well as the patient (clinical data). Cadres of provider training are currently being redefined toward a higher standard of care; therefore, over time, this issue of a minimum standard should be resolved.

### Strengths and limitations

No studies to date have assessed the caseload of burn injuries from the pre-hospital perspective within South Africa or any other LMIC settings. The data collected spanned the whole of the Western Cape Province and captured a window of twelve consecutive months. The size of the study allowed for a provincial investigation of the burden of burns on the operations within WC EMS. Furthermore, no major operational changes have occurred within EMS since this study was conducted. These data may be generalisable to other middle-income countries including African and Latin American countries with EMS systems and a burden of burns. However, no other literature about pre-hospital burns could be found.

It is likely that some burns, originally reported as something else, were missed. There is no reason to believe additional cases would significantly change the observed differences in reported results (e.g., between rural and urban).

An important factor that could have restricted the study’s findings was that of missing data, an inherent limitation in retrospective studies. This missingness only affects the total number of burns counted, and not the distribution throughout the province. However, missing data within patient report forms was also common; among others, burn size, burn depth, TEWS and triage were also not missing at random, as the omissions were more common in patients that remained on scene. Lack of time was not found to explain missingness; time spent on-scene was more than double for non-transported patients where burn size was not provided than when it was provided. It is likely that these burns were less severe and therefore would not affect hospital or burn unit admissions but may be important for determining time and resource utilisation for burns as well as other traumas. Further investigation is warranted to determine any factors that prohibit or enable information captured by EMS, specifically for burns.

Three time metrics were reported as documented by EMS personnel and show similarities (with other traumas) and differences (in urban and rural, P1 and P2) between calls. While potentially unreliable, this is the current practice in this EMS system.

Areas classified as rural in this study may have many of the amenities of an urban or peri-urban setting. However, regardless of the accuracy of the designation, what these areas lack are central hospitals and dedicated burn units: these facilities are only located in the City of Cape Town.

## Conclusions

Burns treated by EMS in this setting form a small part of the overall cases. While priority response time targets were not met in urban areas, they appear to be on par with other trauma cases. Interestingly, while EMS personnel did not classify most burns as urgent, the injuries frequently met the burn centre referral criteria. Due to this, the implications of SATS and the referral criteria for burns have been questioned. Finally, attempts should be made to better understand burn care in EMS from the perspectives of pre-hospital healthcare providers, specifically regarding perceptions of care assessment, training and documentation.

## Abbreviations

ALS: Advanced life support
BLS: Basic life support
EMS: Emergency medical services
ILS: Intermediate life support
IQR: Interquartile range
LMIC: Low- or middle-income country
SATS: South African triage scale
TEWS: Trauma early warning score
WCG: Western Cape government

## References

[1] Goosen J, Bowley DM, Degiannis E, Plani F (2003). Trauma care systems in South Africa. Injury.

[2] World Health Assembly. Health systems: emergency-care systems. WHA60.22. Geneva: Sixtieth World Health Assembly; 2007 p. 21–3.

[3] Mock C, Lormand J, Goosen J, Joshipura M, Peden M (2004). Guidelines for essential trauma care.

[4] Kobusingye OC, Hyder AA, Bishai D, Joshipura ERH, Mock C. Emergency medical services. In: Jamison D, Breman J, Measham A, et al., editors. Disease control priorities in developing countries. 2nd ed. Washington D.C: World Bank; 1996. p. 1261–79. Available from: http://www.ncbi.nlm.nih.gov/books/NBK11744/.

[5] Brysiewicz P, Bruce J (2008). Emergency nursing in South Africa. Int Emerg Nurs.

[6] Western Cape Government: Health. Healthcare 2030: the road to wellness. Cape Town; 2013.

[7] World Health Organization. A WHO plan for burn prevention and care. Geneva: World Health Organization; 2008. 10.2471/BLT.08.059733 PMC2755313 19876549

[8] Minnie L, Goodman S, Wallis L. Exposure to daily trauma: the experiences and coping mechanism of Emergency Medical Personnel. A cross-sectional study. African J Emerg Med. 2015;5(1):12–8. Available from: http://linkinghub.elsevier.com/retrieve/pii/S2211419X14001426.

[9] WHO | The global burden of disease: 2004 update. World Health Organization. Available from: http://www.who.int/healthinfo/global_burden_disease/2004_report_update/en/#.UwO8cp1-38w.mendeley. [cited 2014 Feb 18]

[10] van Niekerk A, Lau U. Third review: crime, violence and injury in South Africa: 21st century solutions for child safety. 2012. Available from: http://www.mrc.ac.za/crime/review.htm.

[11] Blom L, van Niekerk A, Laflamme L. Epidemiology of fatal burns in rural South Africa: a mortuary register-based study from Mpumalanga Province. Burns. Elsevier Ltd and International Society of Burns Injuries; 2011;37(8):1394–402. Available from: http://www.ncbi.nlm.nih.gov/pubmed/21855219. [cited 2014 Jul 20]. 10.1016/j.burns.2011.07.014 21855219

[12] Atiyeh B, Masellis A, Conte C. Optimizing burn treatment in developing low-and middle-income countries with limited health care resources (part 1). Ann Burns Fire Disasters. 2009;22(September):189–95. Available from: http://www.pubmedcentral.nih.gov/articlerender.fcgi?artid=3188182&tool=pmcentrez&rendertype=abstract. PMC3188182 21991180

[13] Statistics South Africa. Mid-year population estimates-2013. 2014. Available from: www.statssa.gov.za.

[14] Statistics South Africa. Census 2011 Municipal report-Western Cape. Pretoria: Statistics South Africa; 2012.

[15] Statistics South Africa. Metropolitan municipality. Available from: http://www.statssa.gov.za/?page_id=993&id=city-of-cape-town-municipality. [cited 2014 Aug 14]

[16] Twomey M, Wallis LA, Thompson M Lou, Myers JE. The South African Triage Scale (adult version) provides reliable acuity ratings. Int Emerg Nurs [Internet]. 2012;20(3):142–50. Available from: http://www.ncbi.nlm.nih.gov/pubmed/22726946. [cited 2014 Dec 6] 10.1016/j.ienj.2011.08.002 22726946

[17] Wallis LA, Gottschalk SB, Wood D, Bruijns S, de Vries S, Balfour C (2006). The Cape Triage Score—a triage system for South Africa. South African Med J.

[18] Medal.org. Triage Early Warning System (TEWS) for adults. Available from: http://www.mymedal.org/index.php?n=Military.290135. [cited 2016 Dec 9]

[19] Karpelowsky JS, Wallis L, Madaree A, Rode H. South African burn society burn stabilisation protocol. South African Med J. 2007;97(8):574–7. Available from: http://www.ncbi.nlm.nih.gov/pubmed/17966146. 17966146

[20] Maritz D, Wallis L, Van Der Merwe E, Nel D (2012). The aetiology of adult burns in the Western Cape, South Africa. Burns.

[21] Rogers AD, Allorto NL, Adams S, Adams KG, Hudson DA, Rode H. Isn’t it time for a cadaver skin bank in South Africa? Ann Burns Fire Disasters.2013;XXVI(3):142–6. PMC3917142 24563640

[22] MacFarlane C, Benn C (2003). Evaluation of emergency medical services systems: a classification to assist in determination of indicators. Emerg Med J.

[23] Stein C, Wallis L, Adetunji O (2015). Meeting national response time targets for priority 1 incidents in an urban emergency medical services system in South Africa: more ambulances won’t help. South African Med J.

[24] Chopra M, Kettle H, Wilkinson D, Stirling S (1997). Paediatric burns in a rural South African district hospital. South African Med J.

[25] Sampalis J, Lavoie A, Williams J, Mulder D, Kalina M (1993). Impact of on-site care, prehospital time, and level of in-hospital care on survival in severely injured patients. J Truama.

[26] Suffla S, Van Niekerk A, Duncan N (2004). Crime, violence and injury prevention in South Africa: developments and challenges.

[27] Gottschalk SB, Wood D, DeVries S, Wallis LA, Bruijns S. The Cape Triage Score: a new triage system South Africa. Proposal from the Cape Triage Group. Emerg Med J. 2006;23(2):149–53. Available from: http://www.pubmedcentral.nih.gov/articlerender.fcgi?artid=2564045&tool=pmcentrez&rendertype=abstract. 10.1136/emj.2005.028332 PMC2564045 16439753

[28] Bell N, Simons R, Hameed SM, Schuurman N, Wheeler S (2012). Does direct transport to provincial burn centres improve outcomes? A spatial epidemiology of severe burn injury in British Columbia, 2001–2006. Can J Surg.

